# A systematic review of the occurrence of *Hyalomma* ticks associated with birds migrating between Africa and the Northern Hemisphere

**DOI:** 10.1186/s13071-025-07222-y

**Published:** 2026-01-09

**Authors:** Ruobing Zhou, Panjun Gao, Jacopo G. Cecere, Diego Rubolini, Marion Koopmans, Elisa Mancuso, Henk van der Jeugd, Reina S. Sikkema, Sara Epis, Federica Monaco, Simona Imperio, Qiyong Liu, Hein Sprong, Thomas Krafft

**Affiliations:** 1 Department of Health, Ethics & Society, CAPHRI Care and Public Health Research Institute, Faculty of Health, Medicine and Life Sciences, Maastricht University, Maastricht, The Netherlands; 2 Key Laboratory of Carcinogenesis and Translational Research (Ministry of Education), National Drug Clinical Trial Center, Peking University Cancer Hospital & Institute, Beijing, 100142 China; 3 Area Avifauna Migratrice, Istituto Superiore per la Protezione e la Ricerca Ambientale (ISPRA), Ozzano dell’Emilia (BO), Italy; 4 Dipartimento di Scienze e Politiche Ambientali, Università degli Studi di Milano, Milan, Italy; 5 Department of Viroscience, Erasmus MC, Rotterdam, The Netherlands; 6 Unità Arbo, Hanta e virus emergenti, Dipartimento Malattie Infettive, Istituto Superiore di Sanità, Viale Regina Elena 299, 00161 Rome, Italy; 7 Vogeltrekstation-Netherlands Institute of Ecology NIOO-KNAW, Wageningen, The Netherlands; 8 Dipartimento di Bioscienze, Università degli Studi di Milano, Milan, Italy; 9 Diagnostica e Sorveglianza Malattie Esotiche, Istituto Zooprofilattico Sperimentale dell’Abruzzo e del Molise “G. Caporale”, 64100 Teramo, Italy; 10 National Key Laboratory of Intelligent Tracking and Forecasting for Infectious Diseases, National Institute for Communicable Disease Control and Prevention, Chinese Center for Disease Control and Prevention, Beijing, China; 11 Centre for Zoonoses and Environmental Microbiology, Netherlands National Institute for Public Health and the Environment (RIVM), Bilthoven, The Netherlands

**Keywords:** Migratory birds, CCHF, *Hyalomma* ticks

## Abstract

**Background:**

Crimean-Congo haemorrhagic fever (CCHF) is a tick-borne disease endemic to Africa, Southern Europe, and Western Asia. Its main vectors, *Hyalomma* ticks, can spread to and possibly establish populations in non-endemic regions via migratory birds.

**Methods:**

We summarized the association between migratory birds and *Hyalomma* ticks by analysing spatial and temporal patterns in tick prevalence and infestation intensity on migratory birds through a systematic review of studies conducted in Europe, Western Asia, and Northern Africa between 1954 and 2022.

**Results:**

We reviewed 37 studies and retrieved data from one additional unpublished datasets. Overall, we collected data on the occurrence of 3876 ticks, most of which were in immature life stages, from 1553 individuals of 75 migratory bird species. The prevalence of ticks from both the *Hyalomma* genus and *Hyalomma marginatum* complex ticks *(H. marginatum* and *H. rufipes*) on migrating birds declined significantly with increasing latitude of the sampling sites in spring. Additionally, we found that the infestation intensity of both tick groups on migratory birds was significantly higher among intra-Palearctic migrants than in Afro-Palearctic migrants.

**Conclusions:**

This review underscores the role of migratory bird species in spreading *Hyalomma* ticks across the Northern Hemisphere and highlights their potential as an early warning system for CCHF outbreaks. This study demonstrates the need to integrate migratory birds into CCHF surveillance systems as sentinels of *Hyalomma* tick dispersal, providing a reference framework for monitoring tick-borne risks along avian migration routes.

**Graphical Abstract:**

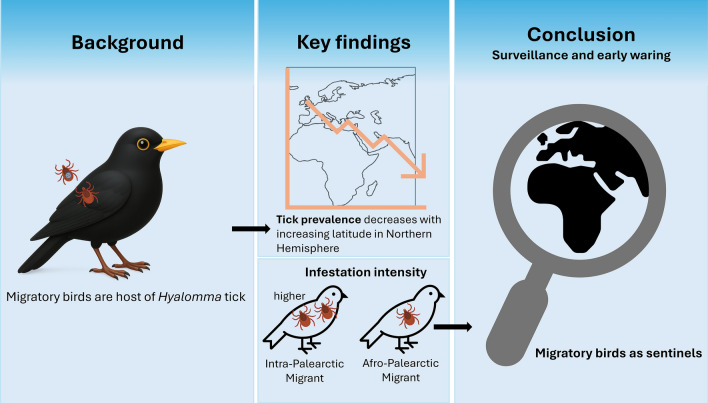

**Supplementary Information:**

The online version contains supplementary material available at 10.1186/s13071-025-07222-y.

## Background

Ticks are currently considered second only to mosquitoes as vectors of human infectious diseases worldwide [1]. They act not only as vectors but also as reservoirs for a wide range of pathogens, capable of transmitting numerous diseases such as Lyme disease, babesiosis, anaplasmosis, and tick-borne encephalitis [2]. Among viral diseases, Crimean-Congo haemorrhagic fever (CCHF) is among the most widespread and lethal tick-borne zoonoses. Endemic across Africa, the Balkans, the Middle East, and Asia, CCHF is caused by the Crimean-Congo haemorrhagic fever virus (CCHFV), a member of the *Orthonairovirus* genus in the Nairoviridae family. The virus persists in natural ecosystems through a tick-vertebrate-tick transmission cycle, with ticks serving as both biological vectors and reservoirs [3].

Ticks can transmit CCHFV through multiple routes, including vertical transmission between generations, trans-stadial transmission between developmental stages, sexual transmission from males to females, and co-feeding transmission between ticks feeding closely on the same non-viraemic host [4]. Transmission to humans occurs via tick bites or direct contact with infected blood and tissues. While CCHFV has been detected in several tick genera, including *Amblyomma*, *Rhipicephalus*, and *Dermacentor*, *Hyalomma* ticks (notably *Hyalomma marginatum* and *H. rufipes*) are considered the predominant competent vectors in supporting the circulation of the virus in natural foci [4]. Typical vertebrate hosts for *Hyalomma* differ by tick life stage: adults are wild and domestic ungulates, whereas immatures are rodents, lagomorphs, and birds [5]. Although mammals and only two species of birds (ostrich [*Struthio camelus*] and guineafowl [*Numida meleagris*]) are known to contribute to virus amplification [6, 7], they often remain asymptomatic when infested, while humans can fall severely ill post-infection, with up to a 40% case fatality rate [8, 9].

In recent years, records of *Hyalomma* ticks have become more common in Eurasia, with some specimens noted outside their historically known distribution range [10]. Such records outside the native distribution range are primarily attributed to the long-range movements of avian host populations, as ticks can spread across long distances via migratory birds on which they feed, covering regional and intercontinental distances in a short time [11, 12]. Migratory birds that breed in Europe undertake long journeys twice a year—during the boreal spring and autumn—between breeding and non-breeding grounds in the Mediterranean Basin (Intra-Palearctic flyway) or in sub-Saharan Africa (Afro-Palearctic flyway) [12–15]. Along the way, these birds may serve as feeding hosts for the immature stages of *Hyalomma* ticks, facilitating the crossing of geographical barriers such as seas and deserts and promoting long-distance dispersal of their ectoparasite community [16–18].

Studies conducted in Europe and North Africa have underscored the involvement of migratory birds in the dispersal of *Hyalomma* ticks, with CCHFV detected in ticks carried by migratory birds [19–22]. However, knowledge of ticks and their relationship with migratory birds is patchy. We performed a systematic review to summarize the association between migratory birds and *Hyalomma* ticks, specifically focusing on spatial and temporal patterns in the prevalence and intensity of infestation of these ticks on migratory birds, with the aim of (i) providing a comprehensive overview of the potential spread patterns of competent vectors for CCHFV and (ii) developing a surveillance framework for timely detection of CCHF outbreak risk in non-endemic regions.

## Methods

### Subject definition, search strategy and search terms

A systematic literature search was completed on May 16, 2024, following the guidelines of the Preferred Reporting Items for Systematic Reviews and Meta-Analyses (PRISMA 2020 statement).

We focused on *Hyalomma* ticks, which are considered the main competent vectors of CCHF based on laboratory studies and field observations [4], and examined their occurrence on avian hosts identified as regular migrants in the target regions [18, 20, 23–33].

The PubMed (PMD), Web of Science (WoS), and Scopus databases were used for a literature search, with no restriction on the time period of included studies to encompass as many relevant articles as possible. All records gathered from the databases were included in the screening process, and duplicate articles were deleted. Unpublished data collected by co-authors in The Netherlands were considered a single study.

The search terms were grouped into three categories: migratory birds, *Hyalomma* ticks, and Crimean-Congo haemorrhagic fever (CCHF). The search query was constructed to retrieve literature that included either the association of migratory birds with *Hyalomma* ticks or their association with CCHF and was limited to the fields title/abstract. All search terms are listed in Additional file 1: Table S1.

### Inclusion criteria and data collection

We considered English-language articles that provided full-text access and focused on research on ticks and birds in Europe, Africa, and Western Asia. To ensure comprehensive coverage, we reviewed the references of all included studies to identify additional studies that were missed in the previous search. The screening process was performed independently by two coauthors, and the results were then compared.

For each study, we recorded the following information: (i) avian host species, (ii) sample site, (iii) sample time (reported as boreal astronomic season), (iv) sample size (number of examined avian host), (v) tick species, (vi) tick stage, (vii) number of *Hyalomma* tick-infested birds, and (viii) number of *Hyalomma* ticks. For articles published within the last 10 years that met the inclusion criteria but lacked complete data, we contacted the corresponding authors to request detailed information, thereby expanding our dataset (Additional file 2: Table S2).

### Data analysis

Bird host species' common and scientific names followed the HBW/BirdLife Taxonomic Checklist [34]. We classified bird species by their migratory strategy as intra-Palearctic or Afro-Palearctic migrants following BirdLife International [13]. Data for seven species were excluded: two (citrine wagtail [*Motacilla citreola*] and brown shrike [*Lanius cristatus]*) were Eastern Palearctic migrants, and five could not be univocally classified because their non-breeding distributions span two biogeographic realms—kentish plover (*Charadrius alexandrinus*), Eurasian buzzard (*Buteo buteo*), red-breasted flycatcher (*Ficedula parva*), isabelline wheatear (*Oenanthe isabellina*), and black-headed bunting (*Emberiza melanocephala*). *Hyalomma marginatum* and* H. rufipes* are the primary competent vectors of CCHFV [4]. These two taxa have long been regarded as belonging to the same species complex and were separated only recently, and their distinction based on morphology is challenging [35]. Moreover, the morphological identification of engorged larvae and nymphs is often unfeasible, and they are reported as *Hyalomma* spp. [36]. We therefore decided to combine observations of *H. marginatum* and *H. rufipes* into a single category, *Hyalomma marginatum* complex ticks. Additionally, we considered all recorded *Hyalomma* ticks, regardless of species, as a *Hyalomma* species category. The sampling season was determined from descriptions in the articles. For studies that clearly defined their sampling season, the reported season was included in the final dataset. For those lacking explicit information, we inferred the season from the sampling dates and categorized them as follows: spring (March–May), summer (June–August), and autumn (September–November). When a study spanned > 2 years, the sampling year was determined as the mean year, calculated as the average of all included years. The coordinates of sampling sites were extracted from the articles or retrieved from geographical maps. If a study involved multiple locations but did not report data separately, the mean coordinates were calculated and used.

We investigated the spatial and temporal patterns of *Hyalomma* tick prevalence and intensity of tick infestation using generalized linear mixed models (GLMMs) fitted through the ‘*glmmTMB’* package [37, 38]. Separate models were fitted for *H. marginatum* complex ticks and *Hyalomma* spp. to both prevalence and infestation intensity data.

Tick prevalence was defined as the number of individual birds infested by at least one tick, divided by the total number of individual birds examined, per species and study [39]. To construct the response variable, we used the *cbind()* function to combine the number of infested individual birds and the number of non-infested birds. Next, we fitted a binomial GLMMs with a logit link function [37, 38]. Sampling year, latitude, longitude, and migratory strategy (a two-level factor) were included as fixed factors, while bird species and study identity (data source) were included as random effects. We further included an observation level random effect to account for overdispersion. To ensure reliable prevalence estimates, only avian host species with at least 20 individuals examined in each study were included in this analysis [40]. Autumn data were excluded because sampling was too sparse and the sample size was small, which would have compromised model reliability. Interaction effects between flyway and latitude/longitude were included in initial models and removed if not significant.

1 $${\text{Tick prevalence}} = \frac{{\text{number of individual birds infested by at least one tick}}}{{\text{total number of individual birds examined}}}$$

The intensity of tick infestation was defined as the total number of ticks divided by the number of tick-infested individuals of the host species in each study. The mean infestation intensity was calculated for each record, and the median of these intensities across studies was then used in subsequent analyses. To model intensity, we relied on negative binomial GLMMs with a log link function. We included the number of ticks as the response variable, with the ln of the total number of birds examined as an offset; sampling year, latitude, longitude, and flyways as fixed effects, and bird species and study identity as random effects. To mitigate potential biases from small sample sizes in infected host populations, our analysis was restricted to studies documenting infestation in at least five individuals per avian host species. Autumn sampling periods were excluded from the analysis because of limited sampling [41, 42]. Interaction effects between flyway and latitude/longitude were included in the initial models and removed if not significant. Model diagnostics were checked by ‘*DHARMa’* package [43]. All analyses were implemented using R version 4.4.1 [44].

2 $${\text{Intensity of tick infestation}} = \frac{{\text{total number of ticks}}}{{{\text{number of tick}} - {\text{infested individuals of the host species in each study}}}}$$

## Results

### General overview of datasets

**Fig. 1:**
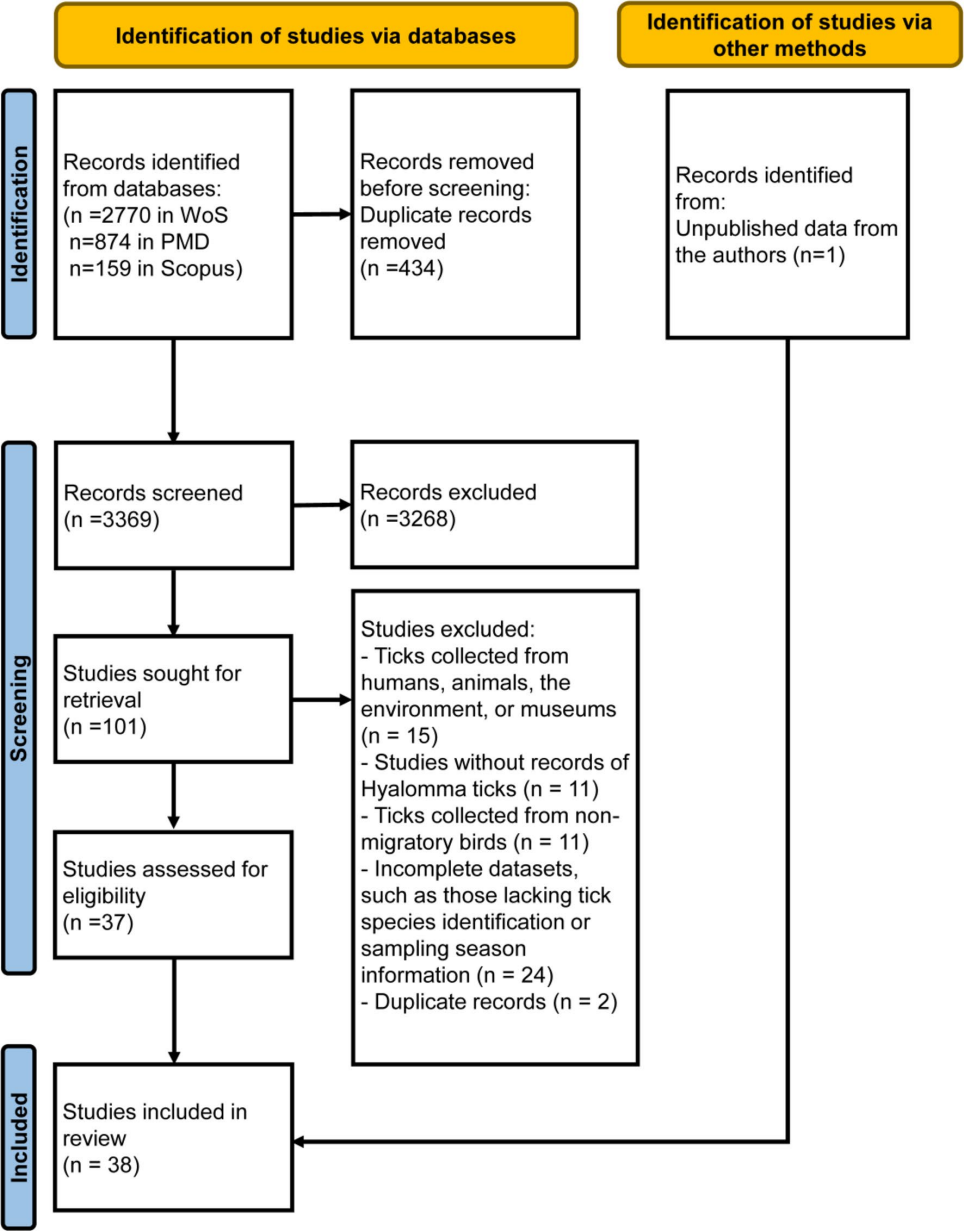
PRISMA flowchart for the study selection procedure

**Fig. 2:**
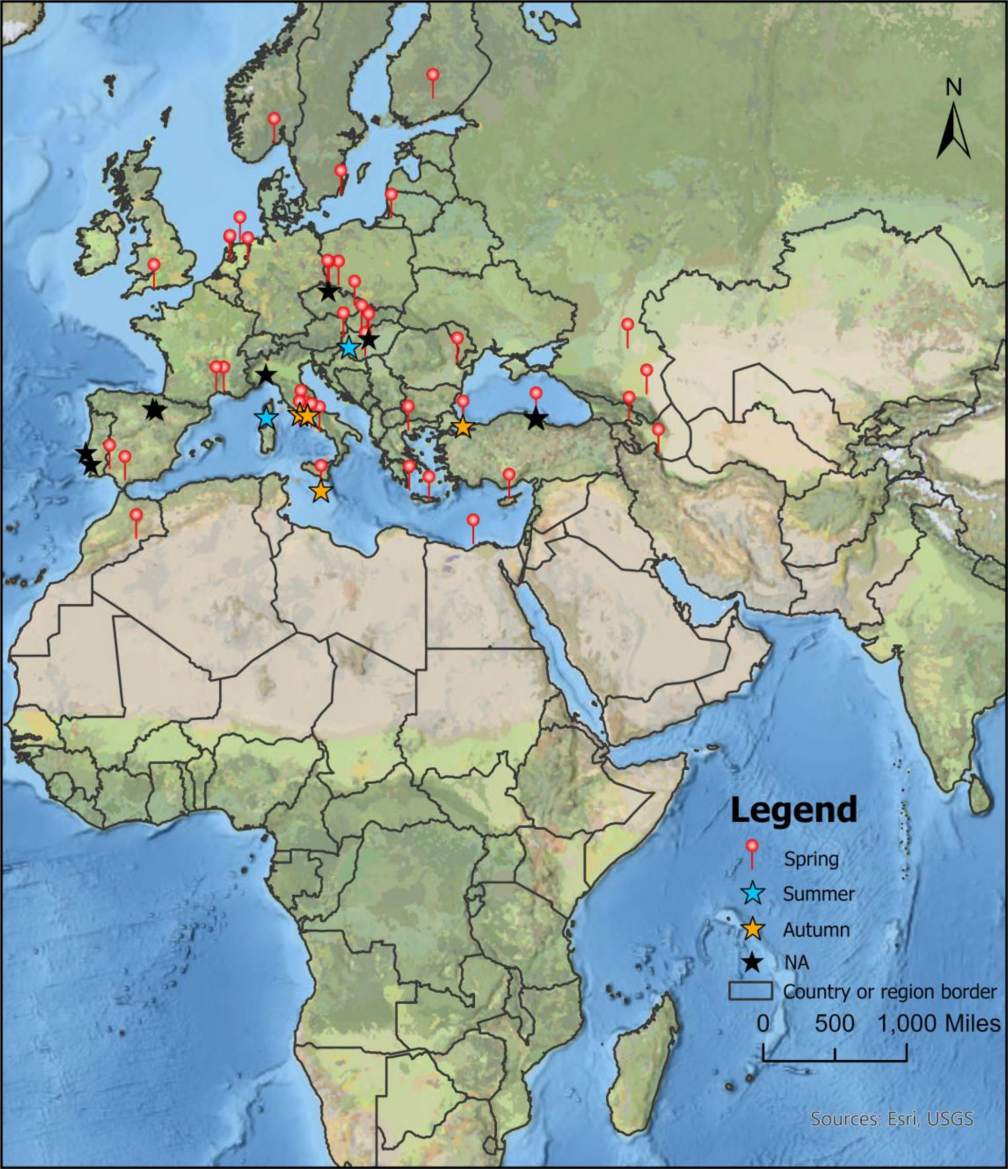
Sampling sites for the studies included in the present review. The red push pin icon represents the spring sampling site, while the orange, blue, and black star icons correspond to the autumn, summer, and unspecified season (NA) sampling sites, respectively. Base layers from (https://hub.arcgis.com/datasets/esri::world-countries-generalized/explore); lines delimiting countries are shown to facilitate map interpretation and do not necessarily represent accepted national boundaries. Terms of use (https://www.esri.com/content/dam/esrisites/en-us/media/legal/product-specific-terms-of-use/e300.pdf)

We identified 3803 bibliographical records through our literature search. After removing duplicate records and screening studies according to the inclusion criteria, 101 articles were eligible for full-text retrieval (Fig. 1). Thirty-seven of the 101 studies contained valid, unduplicated data, all of which were in the English language. Unpublished data provided by the co-authors were considered the 38th study. Studies were conducted in 21 countries across Africa, Asia, and Europe from 1954 to 2022. A total of 3876 *Hyalomma* ticks were collected from 1553 avian hosts (75 species), with 3503 (90.4%), 368 (9.5%), and 5 (0.1%) specimens captured in spring, autumn, and summer, respectively (Fig. 2). Immature stages accounted for > 99% of all specimens.

### *Hyalomma* tick infestation on avian hosts

To calculate the seasonal prevalence of *H. marginatum* complex ticks for each avian host species, we selected studies that examined at least 20 individual avian hosts (see Methods 3.3). The dataset comprised 154 records, representing 84,819 avian hosts from 55 species. Among the hosts examined, 975 were infested with at least one *H. marginatum* complex tick. Table 1 reports tick prevalence for each avian host species by sampling season. Birds were sampled mostly in spring, with far fewer records in autumn (Table 1). The overall prevalence ranges from 0.1% to 21.2% across different species. The highest prevalence of *H. marginatum* complex ticks was observed in the common kestrel (*Falco tinnunculus*; 21.21%) in a study published in 1967. It was followed by the common stonechat (*Saxicola torquatus*) and song thrush (*Turdus philomelos*) with prevalences of 11.8% and 11.0%, respectively. The remaining bird species had an overall prevalence < 10%.

**Table 1:** Prevalence of *Hyalomma marginatum* complex ticks per avian host species (number of infested birds/total examined birds); only data for studies where at least 20 individuals were examined are shown

Species	Spring	Autumn	Spring or autumn	Overall
*Falco tinnunculus*	21.2% (7/33)	NA	NA	21.2% (7/33)
*Saxicola torquatus*	4% (3/75)	33.3% (9/27)	NA	11.8% (12/102)
*Turdus philomelos*	11% (61/555)	NA	NA	11% (61/555)
*Galerida cristata*	NA	9.1% (2/22)	NA	9.1% (2/22)
*Saxicola rubetra*	6.5% (83/1276)	NA	NA	6.5% (83/1276)
*Otus scops*	6.4% (7/109)	NA	NA	6.4% (7/109)
*Oenanthe pleschanka*	6.3% (18/284)	NA	NA	6.3% (18/284)
*Anthus trivialis*	5.8% (15/258)	NA	NA	5.8% (15/258)
*Acrocephalus palustris*	2.9% (1/35)	8.3% (2/24)	NA	5.1% (3/59)
*Acrocephalus arundinaceus*	4.2% (6/143)	NA	NA	4.2% (6/143)
*Phylloscopus sibilatrix*	4.1% (96/2333)	NA	NA	4.1% (96/2333)
*Prunella modularis*	4% (1/25)	NA	NA	4% (1/25)
*Motacilla alba*	NA	3.9% (9/230)	NA	3.9% (9/230)
*Phoenicurus phoenicurus*	3.9% (112/2866)	1.1% (6/547)	NA	3.5% (118/3413)
*Luscinia luscinia*	3.5% (4/114)	NA	NA	3.5% (4/114)
*Oenanthe hispanica*	3.2% (3/94)	NA	NA	3.2% (3/94)
*Muscicapa striata*	3.2% (55/1737)	2.4% (1/42)	NA	3.1% (56/1779)
*Carduelis citrinella*	NA	2.9% (1/34)	NA	2.9% (1/34)
*Passer hispaniolensis*	NA	2.9% (1/35)	NA	2.9% (1/35)
*Coracias garrulus*	NA	2.8% (1/36)	NA	2.8% (1/36)
*Chloris chloris*	NA	2.7% (10/364)	NA	2.7% (10/364)
*Curruca communis*	2.7% (136/5099)	NA	NA	2.7% (136/5099)
*Motacilla flava*	5.1% (5/99)	1.4% (3/210)	NA	2.6% (8/309)
*Fringilla coelebs*	2.6% (2/76)	NA	NA	2.6% (2/76)
*Emberiza caesia*	2.4% (1/41)	NA	NA	2.4% (1/41)
*Ficedula hypoleuca*	2.3% (58/2554)	NA	NA	2.3% (58/2554)
*Lanius senator*	2.2% (4/182)	NA	NA	2.2% (4/182)
*Oriolus oriolus*	2.5% (6/238)	1% (1/102)	NA	2.1% (7/340)
*Luscinia megarhynchos*	2.1% (17/814)	NA	NA	2.1% (17/814)
*Curruca melanothorax*	2% (2/101)	NA	NA	2% (2/101)
*Curruca nisoria*	2% (1/51)	NA	NA	2% (1/51)
*Cuculus canorus*	1.7% (1/58)	2.1% (1/47)	NA	1.9% (2/105)
*Carduelis carduelis*	1.9% (1/54)	NA	NA	1.9% (1/54)
*Acrocephalus schoenobaenus*	1.7% (17/1019)	NA	NA	1.7% (17/1019)
*Iduna pallida*	1.7% (6/355)	NA	NA	1.7% (6/355)
*Oenanthe oenanthe*	1.6% (14/854)	NA	NA	1.6% (14/854)
*Emberiza calandra*	1.4% (1/71)	NA	NA	1.4% (1/71)
*Cettia cetti*	NA	NA	1.4% (1/69)	1.4% (1/69)
*Ficedula albicollis*	1.3% (5/390)	NA	NA	1.3% (5/390)
*Hippolais icterina*	1.2% (53/4522)	NA	NA	1.2% (53/4522)
*Lanius collurio*	1.1% (8/741)	NA	NA	1.1% (8/741)
*Locustella luscinioides*	1.1% (1/90)	NA	NA	1.1% (1/90)
*Hirundo rustica*	0.8% (1/133)	NA	NA	0.8% (1/133)
*Acrocephalus scirpaceus*	0.6% (12/1917)	NA	NA	0.6% (12/1917)
*Upupa epops*	0.6% (2/358)	NA	NA	0.6% (2/358)
*Turdus merula*	NA	NA	0.6% (1/175)	0.6% (1/175)
*Phylloscopus trochilus*	0.3% (11/4118)	3.4% (10/295)	NA	0.5% (21/4413)
*Sylvia atricapilla*	0.3% (20/6049)	0.4% (1/255)	NA	0.3% (21/6304)
*Sylvia borin*	0.3% (16/6089)	1.8% (3/166)	NA	0.3% (19/6255)
*Streptopelia turtur*	1.1% (1/88)	0.1% (1/1237)	NA	0.2% (2/1325)
*Curruca hortensis*	0.2% (2/1031)	NA	NA	0.2% (2/1031)
*Curruca curruca*	0.1% (3/5372)	5% (1/20)	NA	0.1% (4/5392)
*Erithacus rubecula*	0.1% (16/19652)	0.7% (7/1041)	NA	0.1% (23/20693)
*Phylloscopus collybita*	0% (1/3606)	4.9% (2/41)	NA	0.1% (3/3647)
*Curruca cantillans*	0.1% (5/4041)	NA	NA	0.1% (5/4041)

Species are sorted by decreasing prevalence

**Fig. 3:**
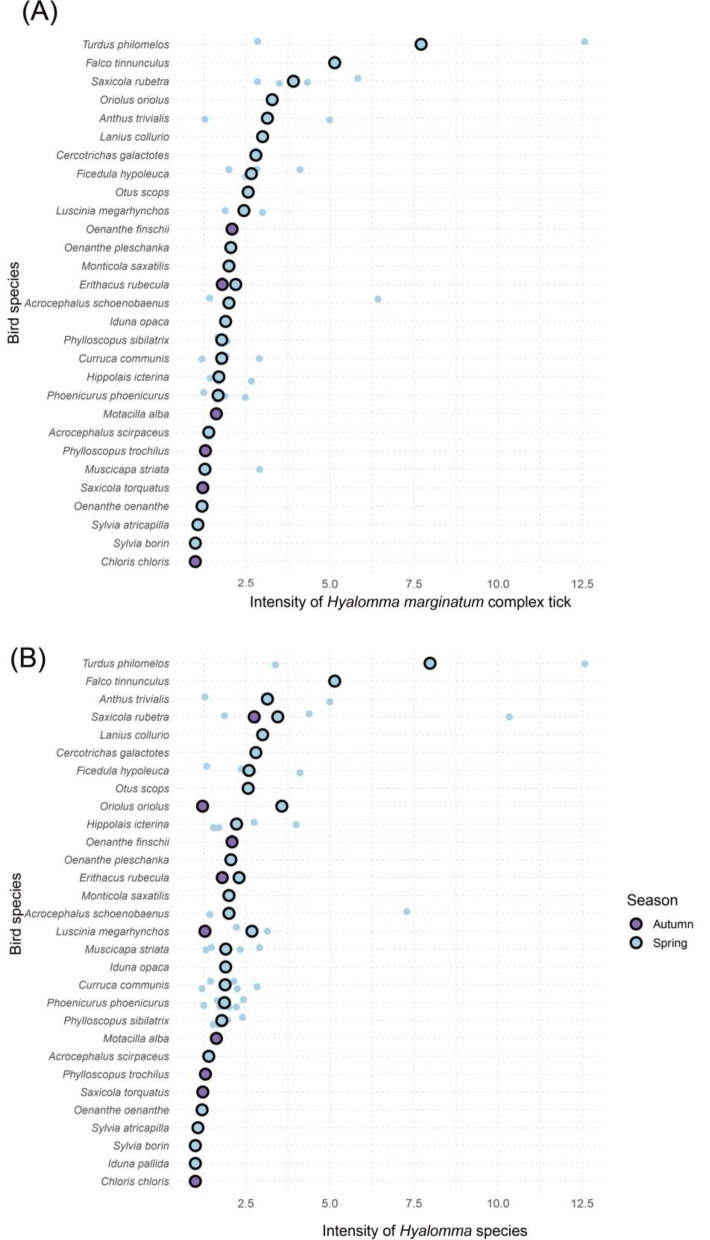
Intensity of *Hyalomma marginatum* complex tick (**A**) and *Hyalomma* species (**B**) infestation on avian host species (number of ticks per infested individual; only for studies with at least five individuals examined; black-bordered points denote median values; open-circle points denote mean values computed for each observation)

To calculate the intensity of *H. marginatum* complex tick infestations for each avian host species, we selected studies with at least five infested individuals per species (see Methods). This dataset contains 870 birds from 29 species infested by at least one *H. marginatum* complex tick. The median intensity of infestation in spring and autumn is presented in Fig. 3A. Median intensity ranged from 1.00 to 7.71 ticks per individual. The highest intensity was observed in the song thrush, followed by the common kestrel at 5.14 ticks per bird. The remaining 27 species exhibited infestation intensities < 4.00 ticks per bird.

We also summarized the prevalence of *Hyalomma* spp. on each avian host species by season. Records with at least 20 examined birds were included. The dataset used to calculate the prevalence of *Hyalomma* spp. comprised 193 records, representing 128,876 avian hosts across 57 species. Of these, 1200 birds were infested with at least one tick. The prevalence of *Hyalomma* spp. ranged from 0.1% to 21.2%, with higher prevalence consistently observed in common kestrel, common stonechat, and song thrush (Additional file 3: Table S3).

The intensity of *Hyalomma* spp. infestation is presented in Fig. 3B. The median intensity varied among avian host species, ranging from 1 to 7.98 ticks per host. The infestation pattern of *Hyalomma* spp. across all avian hosts was similar to that of the *H. marginatum* complex ticks. Two avian host species, the song thrush and common kestrel, exhibited a tick infestation intensity exceeding five ticks per bird.

### Spatial and temporal patterns of tick prevalence and intensity of infestation

**Fig. 4:**
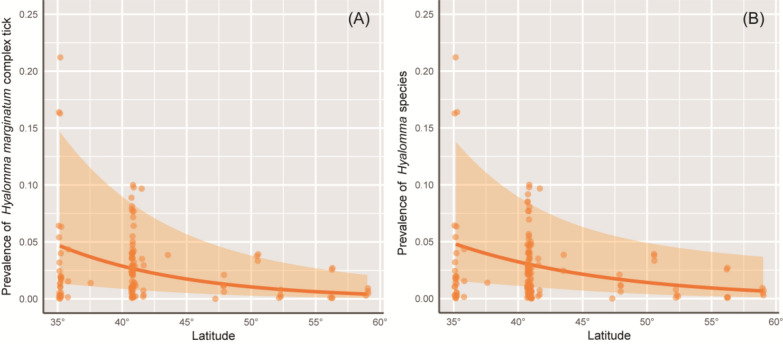
Partial effects of latitude on the prevalence of (**A**) *Hyalomma marginatum* complex ticks and (**B**) *Hyalomma* species

Spring prevalence models were fitted for each tick group. The model for *H. marginatum* complex ticks, based on 152 observations accounting for 1086 infested avian hosts, revealed a significant negative association between prevalence and latitude, with lower prevalence observed at higher latitudes (Fig. 4). A one-degree latitude increase roughly corresponded to a 9.8% drop in *H. marginatum* complex tick infestation (*β* = − 0.103 ± 0.032, *Z* = − 3.232, *P* < 0.01). No significant effects of longitude, sampling year, or flyways were detected (Table 2); the flyways × latitude (*P* = 0.14) and flyways × longitude (*P* = 0.64) interaction effects were non-significant and were excluded. Similarly, the model for *Hyalomma* spp., based on 187 observations involving 1330 infested avian hosts, produced qualitatively similar results, with an estimated 8.2% decrease in *Hyalomma* spp. presence on migratory birds per one-degree increase in latitude (*β* = − 0.085 ± 0.038, *Z* = − 2.213, *P* = 0.027).

**Table 2:** Results of binomial generalized linear mixed models analysing the effects of sampling site, migratory strategy, and sampling year on the prevalence of *Hyalomma marginatum* complex ticks and *Hyalomma* species in spring

Fixed effect	*Hyalomma marginatum* complex ticks				*Hyalomma* species			
	Estimate	SE	*Z*	*P*	Estimate	SE	*Z*	*P*
Latitude	− 0.103	0.032	− 3.232	< 0.01*	− 0.085	0.038	− 2.213	0.027*
Longitude	0.029	0.022	− 1.335	0.182	− 0.137	0.259	− 0.529	0.597
Migratory strategy (intra-Palearctic)	0.203	0.493	0.411	0.681	0.172	0.479	0.359	0.720
Sampling year	0.003	0.013	0.202	0.840	0.246	0.445	0.553	0.580
Random effect	Variance	SD			Variance	SD		
Study identity	0.264	0.513			0.532	0.729		
Bird species	1.357	1.165			1.360	1.167		
Observation level	0.302	0.550			0.313	0.560		

For spring tick infestation intensity models for two *Hyalomma* tick groups, studies documenting infestation in at least five individuals per avian host species were considered. For *H. marginatum* complex ticks, the model included data from 2336 ticks collected from 819 infested birds. In spring, the intensity of *H. marginatum* complex ticks was significantly higher among intra-Palearctic migrants than among Afro-Palearctic ones (RR = 2.38, *P* = 0.040). In terms of *Hyalomma* genus ticks, a total of 2761 ticks from 1004 infested birds were analysed. A similar pattern was observed, with infestation intensity significantly higher among intra-Palearctic migrants than among Afro-Palearctic ones (RR = 2.41, *P* = 0.038) (Table 3).

**Table 3:** Results of negative binomial mixed models analysing the effects of sampling site, flyways, and sampling season on the intensity of *Hyalomma marginatum* complex tick and *Hyalomma* species infestation

Fixed effect	*Hyalomma marginatum* complex ticks				*Hyalomma* species			
	Estimate	SE	*Z*	*P*	Estimate	SE	*Z*	*P*
Latitude	− 0.023	0.036	− 0.641	0.521	− 0.014	0.037	− 0.372	0.710
Longitude	0.010	0.020	0.473	0.636	− 0.002	0.020	− 0.124	0.901
Migratory strategy (intra-Palearctic)	0.868	0.423	2.052	0.040*	0.880	0.425	2.070	0.038*
Sampling year	0.011	0.012	0.946	0.344	0.005	0.012	0.415	0.678
Random effect	Variance	SD			Variance	SD		
Study ID	0.003	0.051			0.010	0.101		
Bird species	0.045	0.212			0.053	0.231		

Autumn was set as the baseline for sampling seasons

## Discussion

Migratory birds can inadvertently spread ticks during their annual migrations, potentially posing public health risks [45]. In Europe, observations of *H. marginatum* and *H. rufipes* have increased in recent years [10, 29, 46, 47]. This increase may reflect either more intense research interest or an increase in *Hyalomma* spp. abundance in the Northern Hemisphere [48], particularly given that *H. rufipes* (generally considered of African origin) has not yet established permanent populations in Eurasia to date [49]. The ongoing increase in *Hyalomma* spp. records may imply a risk of introducing pathogens such as CCHFV to non-endemic areas [20, 50] and could enhance local tick diversity [32, 33, 51]. Most ticks infesting avian hosts are in immature stages. The introduction of immature *Hyalomma* ticks increases the risk of infestations in local vertebrate hosts, such as cattle, horses, and sheep, and may further facilitate CCHFV circulation within animal populations [29].

The present review summarizes the association between migratory birds and their carried *Hyalomma* ticks in Europe, Western Asia, and Northern Africa from articles published between 1954 and 2022. Ticks were analysed separately as two groups: *H. marginatum* complex ticks (*H. marginatum* and *H. rufipes*) and the broader *Hyalomma* spp. Tick prevalence and intensity were calculated for each avian host species, showing variation across different species. Ecological interactions between ticks and their avian hosts are affected by multiple factors, such as avian foraging behaviour, tick host-seeking behaviour, and avian migratory phenotypes [52]. The complete dataset, without any filtering, comprises 75 species from 31 families. Passerine species may serve as sentinel species for monitoring tick dispersal, colonization, and spatial and temporal variation in the risk of tick-borne pathogen outbreaks, such as CCHFV, due to their high abundance and frequent role as tick hosts. Based on the current dataset, monitoring efforts should prioritize avian host species that are commonly observed and exhibit higher *Hyalomma* prevalence or intensity to reach practicality and cost-effectiveness, such as the song thrush, whinchat (*Saxicola rubetra*), and sedge warbler (*Acrocephalus schoenobaenus*). Passerines frequently feed on the ground and are therefore likely to encounter *Hyalomma* ticks [45, 53]. Raptor species were less frequently observed as hosts of *Hyalomma* ticks than passerine species. However, relatively high *Hyalomma* tick prevalence and intensity of infestation were reported for the common kestrel compared with all other avian host species. These data were extracted from a study published in 1968 in Cyprus [54], so the findings have limited generalizability due to the study’s temporal limitations and geographic specificity.

Our quantitative assessment revealed a significant decline in the prevalence of ticks from both *H. marginatum* complex ticks and the *Hyalomma* spp. group on migrating birds with increasing latitude of the sampling sites in spring, while no significant differences in prevalence were observed between the two migratory pathways. This latitudinal gradient may be explained by the geographic distributions of these ticks: *H. marginatum* is widely distributed across North Africa, Asia, and southern Europe [55], whereas *H. rufipes* is predominantly restricted to sub-Saharan Africa [52]. In spring, at lower latitudes, birds forage and prepare for migration, providing opportunities for immature *Hyalomma* ticks to attach. Because most migratory birds divide their journeys into several legs, resting at stopover sites to prepare for the next stage, additional opportunities arise for ticks to attach in these endemic regions. As birds move northward and distance themselves from their African non-breeding grounds and Mediterranean regions [56], fully engorged nymphs may progressively detach from hosts, reducing tick prevalence at higher latitudes.

As for the intensity of tick infestation in spring, our model indicated that the infestation intensity of both tick groups on migratory birds was significantly higher among intra-Palearctic migrants than in Afro-Palearctic ones. This pattern likely reflects ecological differences, including variation in tick abundance within breeding and stopover regions, bird foraging behaviour, climate, and vegetation along these routes [23]. Recent studies confirm the ongoing establishment and expansion of *H. marginatum* populations in southern Europe, driven by host animal movements, anthropogenic activities, and climate change [57, 58]. Although self-sustaining populations of *H. rufipes* have not been documented outside Africa, growing evidence suggests a potential range expansion in the Northern Hemisphere, warranting further investigation within the Palearctic region [59, 60].

Regular monitoring of migratory birds at lower-latitude sites, especially those along the Intra-Palearctic flyway, would provide an efficient and timely approach to assessing the risk and potential pathways of *Hyalomma* tick expansion. Such targeted surveillance exploits the higher prevalence of infestation at these locations and leverages the role of these birds as sentinels for emerging tick introduction and dispersal.

Based on the compiled dataset, most observations were reported in spring, with a few collected in autumn, and only two in summer. The data from summer and autumn were limited and unevenly distributed, both in quantity and in the geographic distribution of sampling sites. This may be attributed to seasonal activity patterns in ticks and sampling bias. Additionally, we applied a threshold in data inclusion while conducting quantitative analysis. As a result, the amount of valid data from autumn and summer was further reduced, preventing a reliable quantitative assessment. Thus, only data from the spring were included in the quantitative analysis. To better understand tick-bird relationships across different seasons, more extensive sampling efforts may be required.

Our study highlights the need for consistent reporting of tick prevalence and infestation intensity data. Although our original dataset comprised information on nearly 6000 *Hyalomma* ticks, one-third of these had to be discarded because of either insufficient data on the number of infested birds or on the sampling season. In studies focusing on tick-borne microorganisms, tick burden on birds, or other tick species, the number of *Hyalomma* ticks or individual hosts examined was often not reported, and individual-level host information was unavailable. Consequently, we used the mean infestation intensity for each record and relied on the median of these values across studies for analyses. Mean intensity is sensitive to the presence of a few highly infested individuals, and using median values helps mitigate the influence of such skewed distributions [61]. We therefore call for more standardized reporting of bird-vector-pathogen interactions, including, but not limited to, details such as sample date, locality (with geographical coordinates), host species, examined host abundance, number of hosts infested with at least one tick, tick species, tick abundance, tick stage, and pathogens detected from tick or host [62, 63]. *Hyalomma marginatum* and *H. rufipes* differ in ecological traits that may influence their respective contributions to the northward dispersal of *Hyalomma* ticks by migratory birds. However, in this article, we referred to *H. marginatum* and *H. rufipes* as *H. marginatum* complex ticks even though the two taxa are regarded as distinct species, because misclassifications have occurred in previous studies relying on morphology-based identification, and the two taxa were not regarded as independent species until only recently [35]. We recommend future studies to be based on molecular tick species identification tools [21, 25, 29, 64].

## Conclusions

The epidemiology of CCHFV in domestic and wild animals, humans, and ticks has been summarized in several reviews to better understand the biology of this pathogen [9, 65–67]. In recent years, increasing attention has been paid to the ecological and health-related implications of associations between migratory birds and hard ticks, particularly in relation to potential tick-borne disease dispersal [52, 68, 69]. Our study builds on this body of work by providing a more comprehensive dataset and insights, specifically on the prevalence and intensity of tick infestations of avian hosts. It helps elucidate the role of avian hosts in the ecology of the CCHF vector. It represents a careful selection of the available literature, highlighting the spatial and temporal patterns in the prevalence and intensity of *Hyalomma* ticks on migratory birds. It underscores the role of migratory birds as sentinel species for the early detection, monitoring, and risk assessment of emerging diseases such as CCHF across Eurasia. We especially recommend implementing effective surveillance protocols for these ticks through broad-scale, long-term monitoring of avian hosts during the pre-breeding migration. We highly advocate for data sharing and transparent reporting of findings to address this emerging global health concern.

## Supplementary Information

Additional file 1.

## Data Availability

Data are provided within the manuscript or supplementary information files.

## Abbreviation

*H**. *: *Hyalomma*
