# Supplementary material for: A systematic review of the occurrence of Hyalomma ticks associated with birds migrating between Africa and the Northern Hemisphere
Source: Parasit Vectors. 2026 Jan 9;19:73. doi: 10.1186/s13071-025-07222-y (PMC12882429; doi:10.1186/s13071-025-07222-y)
Supplement: Supplementary file 1 — Additional file 1. [file 13071_2025_7222_MOESM1_ESM.zip › Additional file 1 Table S1- search term.docx]

| **S1 Table. Search terms** | |
| --- | --- |
| Group | Search terms |
| Migratory bird | migratory bird OR avian OR avian migrant OR bird OR bird of passage OR Flying migrant OR Passage bird OR Seasonal traveler OR Transitory bird OR Traveling bird OR tick-bird OR bird-tick OR migration OR mobile OR Chicken OR Gallus gallus domesticus OR Common blackbird OR Turdus merula OR Common ostrich OR Struthio camelus OR Red-legged partridge OR Alectoris rufa OR Eurasian eagle-owl OR Bubo bubo OR Common wood pigeon OR Columba palumbus OR Passeriformes OR Galliformes OR Strigiformes OR Columbiformes OR Struthioniformes OR Accipitriformes OR Falconiformes OR Coraciiformes OR Bucerotiformes OR Caprimulgiformes OR Barn owl OR Tyto alba OR Barn swallow OR Hirundo rustica OR Black kite OR Milvus migrans OR Black redstart OR Phoenicurus ochruros OR Bluethroat OR Luscinia svecica OR Booted eagle OR Hieraaetus pennatus OR Collared flycatcher OR Ficedula albicollis OR Turdusmerula OR Common buzzard OR Buteo buteo OR Common chaffinch OR Fringilla coelebs OR Common chiffchaff OR Phylloscopus collybita OR Common kestrel OR Falco tinnunculus OR Common kingfisher OR Alcedo atthis OR Common nightingale OR Luscinia megarhynchos OR Common redstart OR Phoenicurus phoenicurus OR Common whitethroat OR Sylvia communis OR Corn bunting OR Emberiza calandra OR Crested lark OR Galerida cristata OR Dunnock OR Prunella modularis OR Eastern olivaceous warbler OR Iduna pallida OR Eastern woodchat shrike OR Lanius senator niloticus OR Eurasian blackcap OR Sylvia atricapilla OR Eurasian blue tit OR Cyanistes caeruleus OR Eurasian eagle-owl OR Eurasian hoopoe OR Upupa epops OR Eurasian jay OR Garrulus glandarius OR Eurasian reed warbler OR Acrocephalus scirpaceus OR Eurasian scops owl OR Otus scops OR Eurasian stonechat OR Saxicola torquatus OR Eurasian tree sparrow OR Passer montanus OR European greenfinch OR Carduelis chloris OR European nightjar OR Caprimulgus europaeus OR European pied flycatcher OR Ficedula hypoleuca OR European robin OR Erithacus rubecula OR European turtle dove OR Streptopelia turtur OR Finsch’s wheatear OR Oenanthe finschii OR Great reed warbler OR Acrocephalus arundinaceus OR Great tit OR Parus major OR House sparrow OR Passer domesticus OR Iberian gray shrike OR Lanius meridionalis OR Lesser kestrel OR Falco naumanni OR Lesser whitethroat OR Sylvia curruca OR Little owl OR Athene noctua OR Marsh warbler OR Acrocephalus palustris OR Mistle trush OR Turdus viscivorus OR Northern goshawk OR Accipiter gentilis OR Ortolan bunting OR Emberiza hortulana OR Red kite OR Milvus milvus OR Rook OR Corvus frugilegus OR Sardinian warbler OR Sylvia melanocephala OR Savi’s warbler OR Locustella luscinioides OR Sedge warbler OR Acrocephalus schoenobaenus OR Song thrush OR Turdus philomelos OR Spanish Sparrow OR Passer hispaniolensis OR Spotted flycatcher OR Muscicapa striata OR Tawny owl OR Strix aluco OR Thrush nightingale OR Luscinia luscinia OR Tree pipit OR Anthus trivialis OR Water pipit OR Anthus spinoletta OR Western jackdaw OR Corvus monedula OR Western olivaceous warbler OR Iduna opaca OR Western yellow wagtail OR Motacilla flava OR Wheatear OR Oenanthe oenanthe OR Whinchat OR Saxicola rubetra OR White wagtail OR Motacilla alba OR Willow warbler OR Phylloscopus trochilus OR Woodchat shrike OR Lanius senator OR Yellowhammer OR Emberiza citrinella Cuculiformes OR Piciformes OR Flaconiformes OR Phoenicurus phoenicuru OR Pied wheatear OR Oenanthe pleschanka OR Red-backed shrike OR Lanius collurio OR Red-throated Pipit OR Anthus cervinus OR Rock thrush OR Monticola saxatilis Sedge warbler OR OrpheanWarbler OR Sylvia hortensis OR Wood warbler OR Phylloscopus sibilatrix OR Hippolais icterina OR Oenanthe hispanica OR Oriolus oriolus OR Pernis apivorus OR Sylvia borin OR Sylvia cantillans |
| Hyalomma tick | tick OR hard tick OR hyalomma tick OR hyalomma OR Ixodes tick OR tick-borne OR Ixodidae OR vector OR Ixodid OR Hyalomma marginatum OR Hyalomma rufipes OR Hyalomma anatolicum OR Hyalomma truncatum OR Hyalomma lusitanicum |
| CCHF | CCHF OR Congo fever OR Congo haemorrhagic fever OR Congo hemorrhagic fever OR Congo-Crimean haemorrhagic fever OR Congo-Crimean hemorrhagic fever OR Crimean fever OR Crimean haemorrhagic fever OR Crimean hemorrhagic fever OR Crimean-Congo haemorrhagic fever OR Crimean-Congo hemorrhagic fever OR Xinjiang haemorrhagic fever OR Xinjiang hemorrhagic fever OR Tick-borne hemorrhagic fever OR Tick-borne haemorrhagic fever OR Hemorrhagic fever virus OR haemorrhagic fever virus OR CHF OR CCHFV OR CHFV OR Congo virus OR Crimean-Congo |
